# The Response Regulator FlmD Regulates Biofilm Formation in *Comamonas testosteroni* through the Transcriptional Activator SoxR

**DOI:** 10.3390/microorganisms10020356

**Published:** 2022-02-04

**Authors:** Yunhao Wang, Zhou Huang, Nan Zhou, Chang Liu, Chengying Jiang, Defeng Li, Shuangjiang Liu

**Affiliations:** 1 State Key Laboratory of Microbial Resources, Institute of Microbiology, Chinese Academy of Sciences, Beijing 100101, China; wangyh@mail.hzau.edu.cn (Y.W.); huangzhou_129@msn.com (Z.H.); joel.c@126.com (N.Z.); LIUC@IM.AC.CN (C.L.); jiangcy@im.ac.cn (C.J.); lidefeng@im.ac.cn (D.L.); 2 College of Life Sciences, University of Chinese Academy of Sciences, Beijing 100049, China; 3 College of Resources and Environment, Huazhong Agricultural University, Wuhan 430070, China; 4 State Key Laboratory of Microbial Biotechnology, Shandong University, Qingdao 266237, China

**Keywords:** biofilm formation, chemosensory pathway, response regulator, efflux pump, transcriptional activator SoxR, *Comamonas testosteroni*

## Abstract

Biofilm formation is a survival strategy by which microorganisms adapt to environmental challenges. It is regulated by various signals, such as the second messenger *c*-di-GMP. We previously found that the Flm chemosensory pathway could respond to chemical signals and regulate biofilm formation. This regulation is independent of *c*-di-GMP. A previous study revealed that the response regulator FlmD is involved in biofilm formation; however, how chemical signals are transmitted downstream of FlmD remained unclear. In the present study, transcriptome analysis and gel shift assay reveal that SoxR, a transcriptional activator of the efflux transporter *acrAB-tolC* operon, mediates the downstream signaling of FlmD. Phosphorylated FlmD interacts with SoxR and disrupts the interaction between SoxR and the *acrAB-tolC* operon. It causes a decrease in the expression of *acrAB-tolC* operon. The downregulation of *acrA*, *acrB*, or *tolC* gene expression results in making less biofilm formation. In conclusion, we identified that the transcription regulator SoxR plays a role in the *c*-di-GMP independent regulation of biofilm formation in *Comamonas testosteroni*.

## 1. Introduction

Biofilm formation is a process in which microbial cells attach and aggregate on surfaces to form a structured multicellular community [1]. Microbial biofilm helps microbial cells protect themselves from environmental stresses and plays key roles in chronic infections, biocorrosion, and bioremediation [2,3,4,5]. Chemotaxis is another strategy for microbial adaptation to new environments. Compared with sessile cells in biofilm, chemotactic cells swim and live in a planktonic state. Chemotaxis enables bacteria to migrate toward favorable niches or to move away from unfavorable ones [6]. Extensive research has revealed that the transition between planktonic and sessile modes of microbial life requires precise and efficient regulation mediated by signaling pathways. Our previous study revealed that two chemosensory pathways coordinate via signal cross-talking for regulation between biofilm formation and chemotaxis in *Comamonas testosteroni* [7].

The chemosensory pathway is a specialized form of two-component system [8]. The key elements of chemosensory pathways are a histidine kinase and a cognate response regulator. However, the histidine kinases of chemosensory pathways receive signals from chemoreceptors and lack signal sensing domains [9]. The chemosensory system of *Escherichia coli* has been well studied as a model; it has five chemoreceptors and only one chemosensory pathway for chemotaxis [10]. Genome analyses have revealed many homologous chemosensory systems, which are often more complex in terms of the number and diversity of chemosensory signaling proteins in other bacterial and archaeal species [11]. Importantly, studies have shown that not all pathways are involved in chemotaxis, but have other functions [9]. Phylogenomic clustering has revealed that chemosensory pathways could be classified into three principal functional classes, namely those associated with flagellar motility (the F class, which can be divided into 17 subclasses, F1–F17), Tfp-based motility (the Tfp class), and alternative (non-motility) cellular functions (the ACF class) [12].

*Comamonas* species are widely distributed in soil, sediments, and garden ponds [13]. Because they miss essential genes for hexose phosphorylation, these species do not grow with glucose as a sole carbon source but metabolize organic acids and aromatic compounds [14,15]. *C. testosteroni* strains play major roles in the bioremediation of contaminated environments and the biological geo-cycling of nutrients. *C. testosteroni* strain CNB-1 has 19 chemoreceptor genes and two chemosensory clusters (*che* and *flm*) [16,17,18]. The *che* cluster belongs to the F7 class and is involved in chemotaxis. The *flm* cluster belongs to the Tfp class but cannot modulate type IV pilus-mediated motility. In a previous study, we demonstrated that *flm* cluster is involved in biofilm formation, and that there is a cross-talk between *che* and *flm* clusters. More interestingly, the cross-talk, which is mediated through the kinase CheA and phosphorylation of response regulator FlmD, could coordinate these two cell processes: chemotaxis and biofilm formation [7].

It is well known that the second messenger 3′,5′-cyclic diguanylic acid (*c*-di-GMP) is a key regulator of biofilm formation. High intracellular *c*-di-GMP concentrations facilitate the transition of bacterial cells from a planktonic state to a sessile one and support biofilm formation [19]. The response regulator WspR of the Wsp pathway, belonging to the ACF class, has a diguanylate cyclase domain (GGDEF) and produces *c*-di-GMP to regulate biofilm formation in the genus *Pseudomonas* [20,21,22]. Unlike WspR, FlmD is a typical single-domain response regulator without an additional GGDEF domain and thus cannot respond by producing *c*-di-GMP. PilH of the Chp/Pil pathway in *P. aeruginosa* is a response regulator homologous to FlmD; it is involved in twitching motility and cAMP-dependent virulence systems (Vfr) [23,24]. However, neither the adenylate cyclase homologue CyaB producing cAMP nor the master transcriptional regulator Vfr could be found in *C. testosteroni*. Thus, the mechanism by which FlmD regulates biofilm formation remained unknown. In this study, we aim to explore the signal transduction of Flm pathway involved in biofilm formation.

## 2. Materials and Methods

### 2.1. Bacterial Strains and Plasmids

The bacterial strains and plasmids used in this study are listed in Table 1. *C. testosteroni* and its mutants were cultivated and maintained at 30 °C in Luria-Bertani (LB) broth or on LB plates with 1.5% (*w*/*v*) agar, and kanamycin was used at 150 μg/mL (broth) or 200 μg/mL (plates) when needed. The *E. coli* strains were cultivated at 37 °C in LB broth or on LB plates with 1.5% (*w*/*v*) agar and kanamycin was used at 50 μg/mL when needed. Genetic disruption and complementation in CNB-1 were conducted using pK18mobSacB and pBBR1MCS-2, respectively (Table 1).

### 2.2. Transcriptome Analysis by RNA-Seq

Samples were cultivated in 6-well plates with duplicates and collected after incubation at 30 °C until reaching the stationary phase. Duplicates were made for each strain. Library construction and sequencing were performed by the Illumina HiSeq platform, and paired-end reads were generated (Novogene, Beijing, China). The sequence reads were mapped, and the expression analysis application was conducted using Bowtie2 and DESeq [27,28]. Genes with an adjusted *p*-value < 0.05 found by DESeq were considered differentially expressed.

### 2.3. Reverse Transcription PCR (RT-PCR) and Quantitative Reverse Transcription PCR (RT-qPCR)

Samples for reverse transcription were cultured in LB at 30 °C and collected after incubation until reaching the stationary phase. Total RNA was extracted by Bacterial RNA Kit (Omega, Norcross, GA, USA). Genomic DNA was removed and cDNA was synthesized by HiFiScript gDNA Removal cDNA Synthesis Kit (CWBIO, Beijing, China). The remaining steps of RT-PCR are the same as PCR. The RT-qPCRs were performed in the LightCycler 480 instrument (Roche, Basel, Switzerland) with KAPA SYBR FAST Universal qPCR Kit (Merck, Kenilworth, IL, USA).

To detect and quantify transcript levels of target genes, primers were designed to amplify about 200 bp region of each gene (RT16sRNA-F: 5′-gcggctgatggcagatta-3′; RT16sRNA-R: 5′-ttacaacccgagggcctt-3′; RT0176-F: 5′-actgaggatgatccgctg-3′; RT0176-R: 5′-cgatctcctccagcgtga-3′; RT0177-F: 5′-cgaggaggccgatacaag-3′; RT0177-R: 5′-cgaaaccagggtggtcag-3′; RT0178-F: 5′-gccgtgatgctggtgttt-3′; RT0178-R: 5′-caggatcgcgttcttgca-3′; RT0179-F: 5′-gctgggagctggatctgt-3′; RT0179-R: 5′-tcgtgtcctgctgattgg-3′). The qPCR run protocol was: pre-incubation (5 min at 95 °C), 40 amplification cycles (10 s at 95 °C; 20 s at 55 °C; 25 s at 72 °C), and melting curve (from 55 to 97 °C). Transcript levels were determined using the comparative CT (threshold cycle) method of relative quantification. All samples have the same 16s rRNA, so the 16S rRNA gene transcripts were established as the internal control reference gene for relative mRNA quantification.

### 2.4. Biofilm Formation Assays and Growth Measurement

Biofilm formation assay was performed as previously described [7]. *C. testosteroni* was cultured in LB overnight, and then cultures were diluted to an OD_600_ = 1.5. 100 μL of the diluted sample was added to a PVC multi-well plate (Corning, Corning, NY, USA), which had been sterilized by UV. Samples were incubated at 30 °C in a humidified incubator for 48 h. Planktonic cells were removed carefully by pipettes, and plates were washed with sterile phosphate buffered saline (PBS) three times. Next, 125 μL of crystal violet (0.1%) were added into the wells and incubated for 30 min. After three washes, 150 μL of 30% acetic acid was added to dissolve the crystal violet and incubated for 10 min. The OD_590_, as the parameter of biofilm biomass, was measured on a multi-well plate reader Victor Nivo (PerkinElmer, Waltham, MA, USA). The growth (OD_600_) of CNB-1 wild-type and mutant strains was measured in LB by using an automated growth curve analysis system (Bioscreen, Turku, Finland).

### 2.5. Genetic Cloning, Overexpression, and Protein Purification

Genes *flmD* and its mutants (*flmDD12K* and *flmDD55A*) were cloned into pET28a and transformed to *E. coli* BL21 (DE3) to generate a C-terminal his-tagged fusion protein. The expression of these genes was induced by the addition of 0.3 mM IPTG at 16 °C for 12 h. Initially, we also used *E. coli* to express and purify the protein SoxR, but failed. Considering SoxR is a transcription factor that might disrupt the normal cellular activity of *E. coli*, *C. testosteroni* replaced *E. coli* as the protein SoxR expression system. Gene *soxR* with a His-tag sequence was cloned into pBBR1MCS-2 and transformed to *C. testosteroni* strain to generate the target protein. There was an interaction between SoxR and FlmD (see the Results section for details), FlmD could be co-purified with SoxR. So, we used *C. testosteroni* CNB-1Δ*flmD* instead of *C. testosteroni* CNB-1 to obtain pure SoxR and exclude the influence from FlmD on subsequent experiments. *C. testosteroni* was cultured in LB overnight, and then cells were harvested for SoxR purification. All proteins were then purified using AKTA FPLC equipped with a HisTrap HP column. Buffer desalting and protein concentration were performed by an Amicon Ultra-15 concentrator (Merck, Kenilworth, IL, USA).

### 2.6. Gel Shift Assay (EMSA)

Gel shift assay was performed for studying gene regulation and determining protein–DNA interactions. DNA probes were prepared by PCR using 5′-FAM-labeled primers: for probe1, probe-F (5′-atggactctcccagaatgaatgcgccc-3′) and probe1-R(5′-gatgtctttctccggggcttgact-3′); for probe2, probe-F (5′- atggactctcccagaatgaatgcgccc-3′) and probe2-R (5′- acctgccaatgcttgtcacaagcc-3′). The SoxR and FlmD/FlmD variant proteins were purified as described above. Thirty-five ng of the labeled probe were incubated with various amounts of purified SoxR and FlmD/FlmD variants for binding rection in 20 μL of binding buffer 20 mM Tris-HCl (pH 7.8), 1 mM MgCl_2_, 40 mM KCl, 2 mM DTT, 0.1 mg/mL bovine serum albumin (BSA), and 5% glycerol] for 20 min at room temperature. The binding mixture was separated from free DNA by electrophoresis through a 6% native polyacrylamide gel at 80 V at 4 °C in 0.5 × TBE running buffer (44.5 mM Tris, 44.5 mM Boric acid, 1 mM EDTA, pH = 8.0). The gels were exposed to chemiluminescence imaging screens for quantitative analysis with Tanon-5200Multi (Tanon, Shanghai, China).

### 2.7. Protein Co-Purification

A protein–protein interaction study was performed as previously described by minor adjustments [29]. Gene *flmD* mutant (*flmDD12K* or *flmDD55A*) with a strep-tag and gene *soxR* with a his-tag were both cloned into pBBR1MCS2 and transformed to *C. testosterni* CNB-1Δ*flmD* for affinity chromatography with StrepTactin Sepharose. The role of FlmD with C-terminal strep-tag is as the bait protein to obtain proteins that interact with FlmD in vivo. Moreover, strep-tag and his-tag could be detected by specific antibodies and were used for protein detection in western blotting. *C. testosteroni* was cultured in LB overnight, and then cells were harvested by centrifugation for 20 min at 3000× *g* at 4 °C and washed twice with the phosphate buffered saline (PBS). The cells were resuspended at a ratio of 10 mL of buffer per 1 g of wet weight in the PBS (pH = 7.0). The cell suspension was lysed twice by using an ultrasonic cell disrupter (SCIENTZ, Ningbo, China). Subsequently, lysates were centrifuged at 10,000× *g* for 15 min to remove insoluble material. The solubilized proteins were incubated with streptavidin beads (Biodragon, Suzhou, China) for 1 h at 4 °C. The beads were pelleted by centrifugation for 1 min at 3000× *g* at 4 °C and washed five times with PBS to remove unbound proteins. Finally, protein binding to streptavidin beads was analyzed by SDS-PAGE and detected by western blotting.

### 2.8. Bacterial Two-Hybrid Assay

The Bacterial two-hybrid system (Agilent, Santa Clara, CA, USA) was used to test the interaction between SoxR and FlmD variants. Plasmid construction and screening were performed according to the manufacturer’s instructions. The pBT and pTRG vectors containing genes of SoxR and FlmD variants were generated. Co- transformants (*E. coli* XL1-Blue strain) containing both pBT and pTRG derivatives were cultured overnight. These cultures were collected and washed by ddH_2_O three times. Three μL bacterial suspensions were inoculated on selective screening medium plate containing 5 mM 3-amino-1,2,4-triazole (3-AT), 12.5 μg/mL streptomycin, 15 μg/mL tetracycline, and 25 μg/mL chloramphenicol at 37 °C for 36 h. The better growth of transformants indicates a stronger interaction.

### 2.9. Sequence Alignment and Analysis

Domains of FlmD were identified and annotated through SMART (SMART: Main page (embl.de, accessed on 29 September 2020) [30]. Promoter and transcription factor (TF) binding sites were predicted through BDGP program (BDGP: Neural Network Promoter Prediction (fruitfly.org, accessed on 29 September 2020) and BPROM program (BPROM—Prediction of bacterial promoters (softberry.com, accessed on 29 September 2020) [31,32]. Multiple sequence alignment was performed by MAFFT online service (MAFFT alignment and NJ/UPGMA phylogeny (cbrc.jp, accessed on 29 September 2020), and the result was visualized by Jalview [33,34].

### 2.10. Statistical Analysis

Statistical analysis was performed using the SigmaPlot 14.0 (Systat Software, Berkshire, UK) program. The biofilm formation was characterized by biofilm biomass with the calculation of means and standard deviations. The statistical significances of differences in biofilm formation between the wild-type and mutant strains were evaluated using Student’s *t*-test (for normal data) or rank-sum test (for nonnormal data), and differences with *p*-value < 0.05 were considered to be statistically significant. The comparative data derived from transcriptome analysis were calculated by DESeq [28], and genes with an adjusted *p*-value < 0.05 were considered differentially expressed.

## 3. Results

### 3.1. Certain Transporter Genes Regulated by FlmD

**Figure 1:**
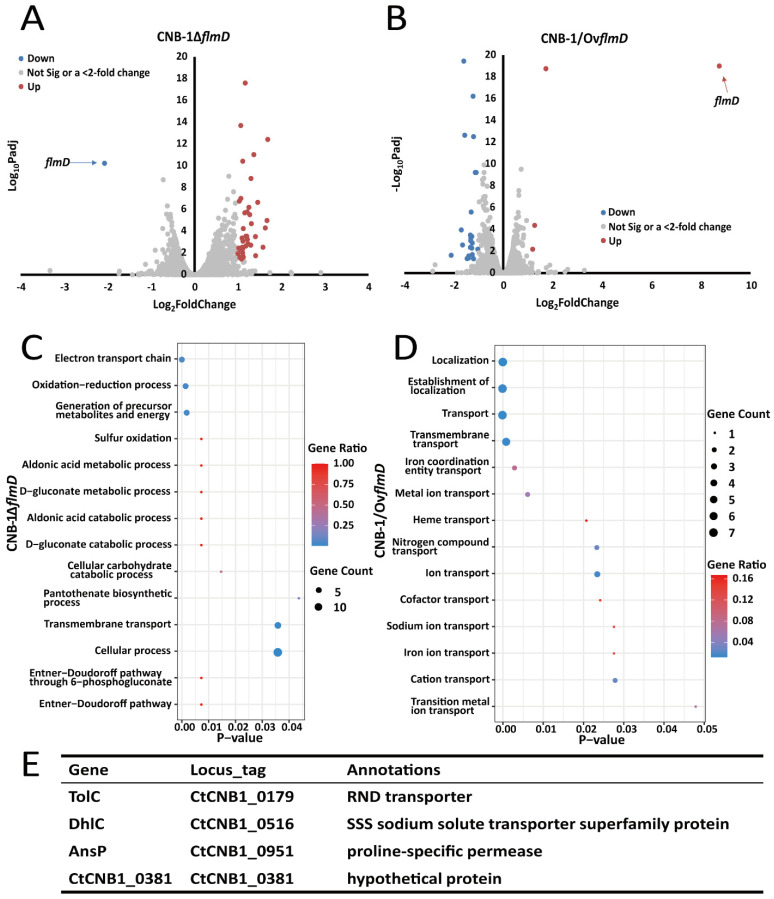
FlmD negatively regulates the transcription of transporters in *C. testosteroni* (**A**); and (**B**) Comparative transcriptome analysis among CNB-1, CNB-1Δ*flmD*, and CNB-1/Ov*flmD* revealed that FlmD negatively regulated a small number of genes. Color scheme: red dots, genes showing significant and >2-fold upregulation; gray dots, genes showing no significant or <2-fold change; and blue dots, genes showing significant and >2-fold downregulation. Padj: *p*-value adjusted for multiple testing with the Benjamini–Hochberg procedure to control the false discovery rate; (**C**) and (**D**) Gene ontology terms in biological process categories associated with material transport were enriched; (**E**) Four genes show significant and >2-fold change in both strains CNB-1Δ*flmD* and CNB-1/Ov*flmD*.

As mentioned above, the Flm pathway regulates biofilm formation in *C. testosteroni*. However, it differs significantly from any known chemosensory pathways mediating biofilm formation. Considering the key role of FlmD in biofilm formation, we performed transcriptome analysis among the wild-type strain (CNB-1), FlmD-deleted mutant strain (CNB-1Δ*flmD*), and FlmD-overexpressing strain (CNB-1/Ov*flmD*) (Figure 1A,B) and attempted to identify the microbial process that was affected by FlmD. Compared with CNB-1, 359 genes in CNB-1Δ*flmD* (Figure 1A) and 307 genes in CNB-1/Ov*flmD* (Figure 1B) showed significant changes (*p* < 0.05). As shown in Table 2, only 39 genes showed >2-fold change in CNB-1Δ*flmD*; these genes were upregulated. Hence, we speculated that FlmD negatively affected the transcription of these genes. Gene ontology (GO) enrichment analysis was performed to understand the functions of these regulated genes in biological processes. The results revealed cellular process and cross-membrane transport to be significantly affected (Figure 1C). In CNB-1/Ov*flmD*, 26 genes exhibited >2-fold change, of which 23 genes were downregulated (Table 3). Consistent with *flmD* deletion, most of these 26 genes are involved in material transport in CNB-1/Ov*flmD* (Figure 1D).

Compared with the total number of genes (4890) in the CNB-1 genome, only 6.3–7.3% of genes (most of which were related to transport systems) had significant changes at the transcription level in the CNB-1Δ*flmD* and CNB-1/Ov*flmD* strains. This result suggests that FlmD is a pathway-specific regulation factor. Further analysis indicated that among these genes, only four genes shared by CNB-1Δ*flmD* and CNB-1/Ov*flmD* exhibited >2-fold change (Figure 1E). The four genes were *tolC* (CtCNB1_0179), *dhlC* (CtCNB1_0516), *ansP* (CtCNB1_0951), and a hypothetical protein-encoding gene (CtCNB1_0381), named by their annotation in the COG database [35]. Sequence analysis showed that all four genes had transmembrane sequences; three genes were annotated as transporters/permeases and one was uncharacterized.

### 3.2. The Effect of TolC on Biofilm Formation

**Figure 2:**
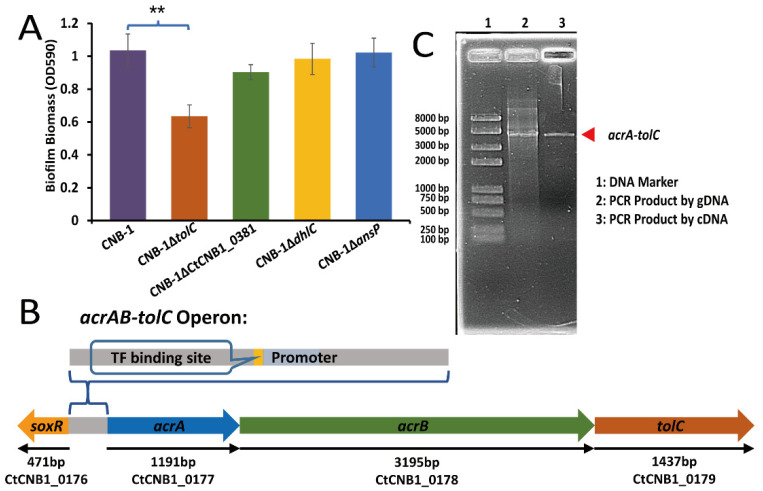
TolC regulates biofilm formation in *C. testosteroni* and belongs to the AcrAB-TolC efflux pump. (**A**) A crystal violet assay measured the effects of *tolC*, *dhlC*, *ansP*, and CtCNB1_0381 on biofilm formation. Only *tolC* deletion significantly reduced biofilm formation; (**B**) Diagram of the *acrAB-tolC* operon; (**C**) *acrA*, *acrB*, and *tolC* were transcribed together to form a single mRNA molecule. The gDNA of CNB-1 was a PCR template in lane 2 (as a positive control), and cDNA was a template in lane 3. Data in panel A are the means and standard deviations from three independent experiments conducted in triplicate. (** *p* < 0.01 with Student’s *t*-test).

Based on the observation that the response regulator FlmD not only affected biofilm formation but also mediated the transcription of some transporter genes, we constructed gene-knockout strains to determine the impact of these transporters on biofilm formation. Compared with the wild-type strain CNB-1, the deletion of *tolC* (CtCNB1_0179) was found to cause a significant biofilm formation defect; however, the other three genes [*dhlC* (CtCNB1_0516), *ansP* (CtCNB1_0951), and a hypothetical protein-encoding gene (CtCNB1_0381)] did not significantly affect biofilm formation (Figure 2A). Several studies have suggested that TolC is an outer membrane channel, a part of the AcrAB-TolC efflux system [36,37]. The AcrAB-TolC efflux pump belongs to the resistance–nodulation–division (RND) family transporters responsible for the efflux of many antibiotics, dyes, and detergents; it is widespread among Gram-negative bacteria [37]. The disruption of the AcrAB-TolC pump reduces biofilm formation [38,39,40], which agrees with our observation in CNB-1.

The periplasmic membrane fusion protein AcrA and the inner membrane RND transporter AcrB, whose corresponding genes (*acrA*, CtCNB1_0177 and *acrB*, CtCNB1_0178) are located upstream of *tolC* in the CNB-1 genome (Figure 2B), are the other two components of the AcrAB-TolC complex [41]. These functionally related genes (*acrA*, *acrB*, and *tolC*) are contiguously located on a stretch of DNA and have an operon-like structure. In addition, these genes are thought to share the same promoter, located upstream of *acrA* (Figure 2B). Therefore, *acrA*, *acrB*, and *tolC* may belong to the same operon. RT-PCR further confirmed this speculation. The result showed that *acrA*, *acrB*, and *tolC* were transcribed together to form a single messenger RNA (mRNA) molecule (Figure 2C). This observation is consistent with the gene transcription profiles of CNB-1Δ*flmD*, both *acrA* and *tolC* had significant changes (more than 2-fold; *p*-value < 0.05) (Table 2).

### 3.3. The Transcriptional Regulation of SoxR on the acrAB-tolC Operon

**Figure 3:**
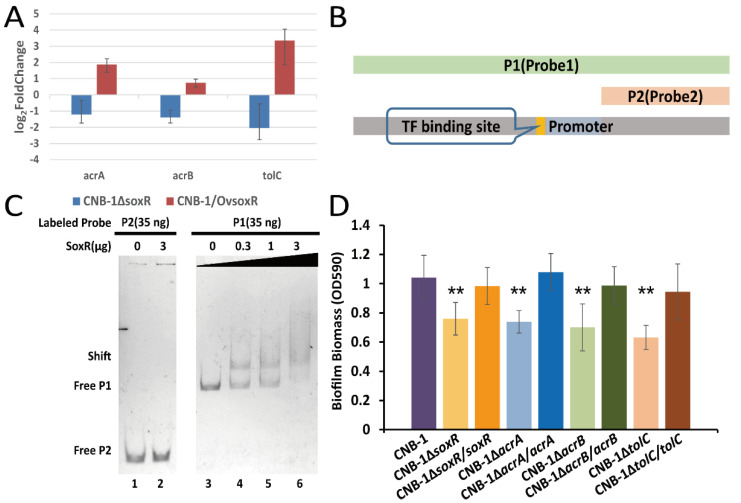
SoxR positively regulates the transcription of the *acrAB-tolC* operon in *C. testosteroni*. (**A**) Transcriptional changes in *acrA*, *acrB*, and *tolC* in *soxR*-deleted and *soxR*-overexpressing strains. (**B**) Diagram of probes for the gel shift assay. (**C**) Determination of the interaction between SoxR and *acrAB-tolC* operon DNA. Probe2 used as the negative control lacked predicted transcription factor binding sites and a promoter. From lanes 3 to 6, the number of Probe1 binding to proteins (shift band) increased with an increase in SoxR concentration. (**D**) Biofilm formation in *acrAB-tolC* operon components-deleted and -complemented mutant strains was assessed using a crystal violet assay. The effect of SoxR on *acrAB-tolC* gene transcription was consistent with that on biofilm formation. Data in panel D are the means and standard deviations from three independent experiments conducted in triplicate. (** *p* < 0.01 with Student’s *t*-test.).

Further sequence analysis of the *acrAB-tolC* operon revealed that a transcription factor (TF) binding site and the transcription factor gene *soxR* (CtCNB1_0176, a redox-sensitive transcriptional activator) are in the upstream sequence of *acrA*, *acrB*, and *tolC* (Figure 2B). The *acrAB-tolC* operon has a similar gene organization to the well-known *lac* operon and *ara* operon [42,43]. We performed RT-qPCR to determine the transcript levels of *acrA*, *acrB*, and *tolC* in *soxR* mutant and wild-type strains. Compared with CNB-1, these genes showed >2-fold change and were downregulated in CNB-1Δ*soxR* (Figure 3A). In CNB-1/Ov*soxR*, these genes were upregulated when *soxR* was overexpressed (Figure 3A). In addition, based on the sequence of the predicted promoter and TF binding site, we designed probes for the gel shift assay (Figure 3B). As expected, we also observed that SoxR is bound to Probe1 DNA, which comprises a predicted promoter and TF binding site (Figure 3C). These results proved SoxR could regulate the transcription of *acrA*, *acrB*, and *tolC*. As SoxR is the transcription regulator of the *acrAB-tolC* operon and TolC is involved in biofilm formation, we then assessed the effect of SoxR on biofilm formation. As shown in Figure 3D, the deletion of *soxR* decreased biofilm formation. Similarly, the disruption of AcrA and AcrB significantly decreased biofilm formation (Figure 3D). Notably, however, the deletion of the corresponding genes did not affect cell growth (Supplementary Figure S1).

### 3.4. FlmD Variants Involved in Phosphorylation

**Figure 4:**
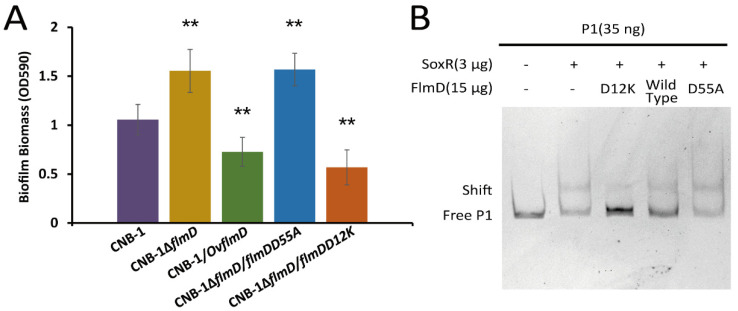
The phosphorylation of FlmD is essential for negative regulation. (**A**) Functional characterization of biofilm formation in *flmD* mutants assessed using crystal violet assay. (**B**) Changes in the interaction between SoxR and the *acrAB-tolC* operon on the addition of FlmD variants to gel shift assay systems are shown. Data in panel A are the means and standard deviations from three independent experiments conducted in triplicate. (** *p* < 0.01 with Student’s *t*-test or rank sum test.).

The phosphorylation of response regulators is important in chemosensory signal transduction. For instance, the binding of phosphorylated CheY (CheY-P) to the flagellar motor protein FliM for inducing a change in the flagellar rotation behavior is the signal output during chemotaxis. In *E. coli*, the CheY(D13K) variant, whose aspartic acid-13 is replaced by lysine, is equivalent to CheY-P [44,45]. The aspartate residue is conserved in response regulators of two-component systems. In addition, the phosphorylation site of response regulators is also conserved. These sites in FlmD are D12 and D55, respectively (Supplementary Figure S2). In order to measure the effect of FlmD phosphorylation, we constructed two variants: (1) FlmD(D12K), as phosphorylation activated variant, and (2) FlmD(D55A), a variant that could not be phosphorylated. As shown in Figure 4A, deletion of *flmD* (CNB-1∆*flmD*) resulted in an upregulation of biofilm formation, whereas overexpression of *flmD* (CNB-1/*OvflmD*) resulted in significant reduction of biofilm formation, indicating FlmD negatively regulates biofilm formation. The FlmD(D55A) variant lacks the phosphor-acceptor site, its related strain CNB-1∆*flmD/flmDD55A* showed enhanced biofilm formation. On the basis of these results, we further concluded that the actual negative regulator is phosphorylated FlmD. FlmD(D12K) is considered a phosphorylation activated variant. The inhibition of FlmD(D12K) on biofilm formation was consistent with expected. Hence, FlmD(D12K) is similar to CheY(D13K) and is equivalent to FlmD-P.

As mentioned above, both FlmD and SoxR could regulate the transcription of the *acrAB-tolC* operon. In order to unravel the relationship between FlmD and SoxR, we assessed the effect of FlmD on SoxR in a gel shift assay system. The result showed the presence of FlmD(D12K) disrupted the interaction between SoxR and probe1 DNA, and the effect of FlmD(D12K) on SoxR was concentration-dependent (Figure 4B and Supplementary Figure S3A). Whereas FlmD(D55A) at any concentration did not affect SoxR (Figure 4B and Supplementary Figure S3B). This finding is consistent with the result that phosphorylated FlmD is the signal output.

### 3.5. The Interaction between FlmD and SoxR

**Figure 5:**
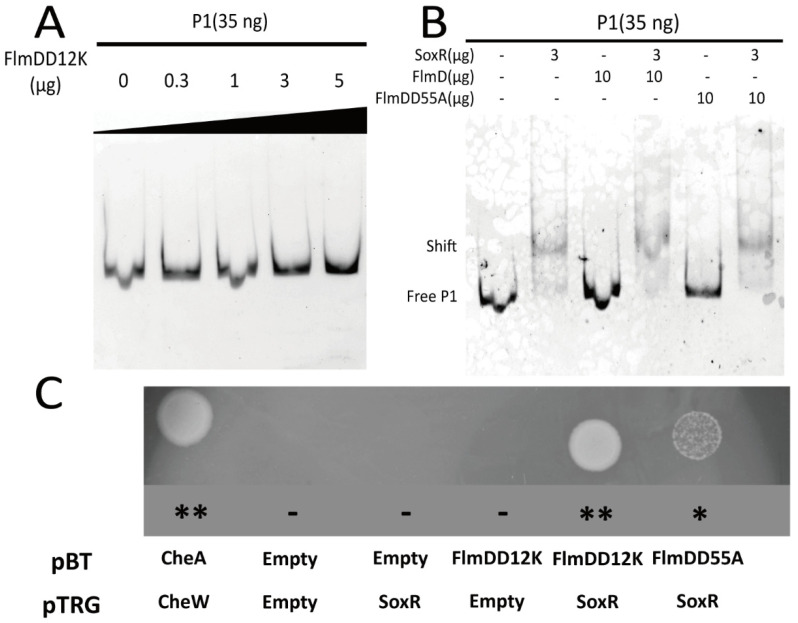
FlmD variant proteins interact with the transcriptional activator SoxR. (**A**,**B**) Interactions between FlmD/FlmD variants and the *acrAB-tolC* operon were assessed using the gel shift assay. Neither FlmDD12K nor FlmDD55A directly interacts with *acrAB-tolC* operon; (**C**) The bacterial two-hybrid system measured interactions between SoxR and FlmD variants. The growth of transformants is shown on selective screening medium plates. Better growth indicates a stronger interaction. The known interaction between CheA and CheW served as positive control. The asterisk and minus indicate the growth status. **: strong; *: weak; -: null.

The present study revealed that FlmD affected the transcriptional regulation of the *acrAB-tolC* operon through the transcription factor SoxR, thereby negatively regulating biofilm formation. FlmD could affect the transcriptional function of SoxR in two ways: by occupying the binding sites on DNA molecules, and by directly interacting with SoxR. However, the gel shift assay revealed that neither FlmD nor its variant FlmD(D12K)/FlmD(D55A) could bind to the *acrAB-tolC* operon (Figure 5A,B). The first way was excluded. To determine the potential interaction between FlmD and SoxR, a two-hybrid assay with FlmD variants FlmD(D12K) and FlmD(D55A) was used. Unexpectedly, SoxR interacted with FlmDD12K as well as FlmDD55A. However, the two-hybrid assay indicates a stronger interaction of SoxR with FlmDD12K than FlmDD55A (Figure 5C). In conclusion, the phosphorylated FlmD (FlmDD12K) had a stronger interaction with SoxR, and this tight binding between the phosphorylated FlmD and SoxR negatively affected the SoxR binding to DNA molecules.

## 4. Discussion

Signal transduction in bacterial and archaeal cells is primarily mediated by one-component, two-component and chemosensory systems [9,12,46]. Our previous study revealed two chemosensory systems (Che and Flm) in *C. testosteroni*. The Che pathway modulates flagellar rotation for chemotaxis, and the Flm pathway modulates biofilm formation. These two processes are coordinated through the kinase CheA and the phosphorylated response regulator FlmD [7]. In the present study, the transcriptome analysis among the wild-type and mutant strains demonstrates that FlmD negatively regulates genes in *acrAB-tolC* operon. Further sequence analysis of the *acrAB-tolC* operon shows SoxR is its transcriptional activator. FlmD, SoxR and AcrAB-TolC efflux pump are all involved in biofilm formation. These results imply both FlmD and SoxR modulate biofilm through the efflux pump. What is the relationship between FlmD and SoxR? The gel shift assay shows phosphorylated FlmD disrupts the interaction between the *acrAB-tolC* operon and SoxR. However, FlmD-P could not bind to the *acrAB-tolC* operon. So, FlmD-P would not occupy the binding sites of DNA molecules. In addition, the two-hybrid assay indicates the phosphorylation of FlmD enhances the interaction between the FlmD and SoxR. The interaction may have a steric effect. Phosphorylated FlmD restricts the SoxR binding to DNA molecules and inhibits the transcription of *acrAB-tolC* operon. Overall, we identified that SoxR, a transcriptional activator of the efflux transporter *acrAB-tolC* operon, is the signal output target of FlmD. Changes in the concentrations of chemical ligands are sensed by chemoreceptors. They transmit signals to FlmA and induce its autophosphorylation. Phosphorylated FlmA transfers the phosphoryl group to cognate response regulator FlmD. Then, FlmD-P disrupts the interaction between SoxR and the *acrAB-tolC* operon, thereby inhibiting the transcription of efflux transporter genes *acrA*, *acrB*, and *tolC*. We also demonstrated that the AcrAB-TolC efflux pump is involved in biofilm formation; this finding is consistent with observations in other bacteria [39,40]. However, how the AcrAB-TolC efflux pump regulates biofilm formation is still unclear. Previous studies have discussed four potential mechanisms (one mechanical mechanism and three chemical mechanisms) by which AcrAB-TolC can regulate biofilm formation [38]. In addition to AcrAB-TolC, TolC is also required for the function of other efflux systems such as EmrAB-TolC and MacAB-TolC [47,48], which exist in *C. testosteroni*. Whether these efflux systems are involved in biofilm formation is unclear.

Numerous studies have reported that the regulatory network of microbial biofilms is very complex [49,50]. At different stages of biofilm development, the regulatory network senses different environmental signals and elicits the corresponding response [51]. Flm belongs to the chemosensory pathway, which can respond to a particular chemical stimulus depending on its associated chemoreceptors. Chemoreceptors detect various chemical signals and transmit this information into chemosensory systems [52]. Based on the gene arrangement in the *flm* cluster, FlmB is the associated chemoreceptor; its sensory repertoire is still unclear. However, our previous study revealed that MCP2201, which belongs to the Che pathway and senses TCA cycle intermediates [53], can transmit signals to the Flm pathway through cross-talk [7]. In other words, TCA cycle intermediates serve as chemical stimuli for regulating biofilm formation through the Flm pathway. The TCA cycle plays a central role in the metabolism of aerobic organisms. Apart from its essential role in energy generation, it provides building blocks for biosynthetic pathways [54]. Furthermore, TCA cycle intermediates are widely distributed in a variety of natural habitats. Consequently, these intermediates are suitable representatives and clues of the current nutritional status of the environment where the bacteria are located. In addition to *Comamonas* species, many other bacteria sense these TCA intermediates for regulating biofilm development. In *Staphylococcus* or *Bacillus* species, citrate can regulate biofilm formation [55,56]. Moreover, the MifS-MifR system of *P. aeruginosa* not only regulates biofilm formation but also senses alpha-ketoglutarate [57].

The Flm pathway of CNB-1 belongs to the Tfp class, but the Flm pathway controls biofilm formation instead of Tfp-based motility. Some chemosensory pathways have been reported that their phylogenomic classes did not match functions. In *P. aeruginosa*, the Che2 pathway (the F7 class) mediates the response to O_2_ stimulus [58]. Tfp-based motility in *M. xanthus* is mediated by Frz and Dif, which belong to ACF and F1, respectively [59]. Recently, the diversity of the functional regulation of chemosensory systems in bacteria has been reviewed [9]. Two hypotheses about the diversity of functional regulation have been proposed: (1) a pathway may switch its output target to obtain other functions, and (2) the function of one pathway may be replaced by another pathway [9,60]. Previously, we tried to explore all genomes in the genus *Comamonas*. Genome data for 11 *Comamonas* species are present in publicly available databases [13]. We found that seven of the 11 *Comamonas* species have *flm* clusters. Interestingly, some clusters of *C. granuli* and *C. badia* have annotated *pilTs*, which encodes a Tfp pilus assembly protein and are involved in twitching motility, and their other components are consistent with those of the *flm* cluster. The presence of *pilT* suggests that these clusters might regulate Tfp-based motility, and yet other components imply that they are related to *flm* cluster, a biofilm formation cluster. As a reasonable hypothesis, *flm* and other clusters might originate from a common ancestor, but a possible evolutionary event that happened in *Comamonas* genus caused the functional divergence between different clusters. SoxR replacing PilT as the signal output target of FlmD or the opposite, endowed these clusters with different functions. The shift from Tfp-based motility to biofilm formation, as exemplified by Flm and its related pathways, is an example of chemosensory pathway obtaining other functions through switching its output target.

## Figures and Tables

**Table 1:** Strains and plasmids used in this study.

Strain/Plasmid	Relevant Genotype or Description	Sources
**Strains**		
*Comamonas testosteroni*		
CNB-1		[25]
CNB-1Δ*flmD*	FlmD (CtCNB1_3988) disrupted in CNB-1	[7]
CNB-1Δ*dhlC*	DhlC (CtCNB1_0516) disrupted in CNB-1	This study
CNB-1Δ*ansP*	AnsP (CtCNB1_0951) disrupted in CNB-1	This study
CNB-1ΔCtCNB1_0381	CtCNB1_0381 disrupted in CNB-1	This study
CNB-1Δ*acrA*	AcrA (CtCNB1_0177) disrupted in CNB-1	This study
CNB-1Δ*acrB*	AcrB (CtCNB1_0178) disrupted in CNB-1	This study
CNB-1Δ*tolC*	TolC (CtCNB1_0179) disrupted in CNB-1	This study
CNB-1Δ*soxR*	SoxR (CtCNB1_0176) disrupted in CNB-1	This study
*Escherichia coli*		
DH5α	F^-^φ80d lacZΔM15 Δ(lacZYA-argF) U169 recA1 endA1 hsdR17(r_K_^-^ m_K_^+^) supE44 λ- thi-1 gyrA96 relA1 phoA; host for DNA manipulations	TransGen
BL21(DE3)	F^-^ ompT hsdS (r_B_^-^ m_B_^-^) gal dcm (DE3)	Novagen
XL1-Blue	MRF’ Kan, glycerol stockb (host strain for propagating pBT and pTRG recombinants)	Stratagene
**Plasmids**		
pBBR1MCS-2	Km^r^, lacPOZ’ broad host vector with R type conjugative origin	[26]
pBBR1MCS2-*flmD*	Carries *flmD* to generate complementation	[7]
pBBR1MCS2-*flmDD55A*	A mutation from an aspartate to an alanine in 55th residue	[7]
pBBR1MCS2-*flmDD12K*	A mutation from an aspartate to a lysine in 12th residue	This study
pBBR1MCS2-*soxR*	Carries *soxR* to generate complementation	This study
pBBR1MCS2-*acrA*	Carries *acrA* to generate complementation	This study
pBBR1MCS2-*acrB*	Carries *acrB* to generate complementation	This study
pBBR1MCS2-*tolC*	Carries *tolC* to generate complementation	This study
pBBR1MCS2-*soxR-his*	pBBR1MCS2 derivative for expression of SoxR	This study
pBBR1MCS2-*flmDD12K-sterp* + *soxR-his*	pBBR1MCS2 derivative for co-purification of SoxR and FlmDD12K	This study
pBBR1MCS2-*flmDD55A-sterp* + *soxR-his*	pBBR1MCS2 derivative for co-purification of SoxR and FlmDD55A	This study
pBBR1MCS2pfer	adds a strong constitutive promoter in pBBR1MCS-2	[7]
pBBR1MCS2pfer-*flmD*	Carries *flmD* to overexpression	[7]
pET28a	Km^r^, bacterial expression vector with a His-tag	Youbio
pET28a-*flmD*	pET28a derivative for expression of FlmD	[7]
pET28a-*flmDD55A*	pET28a derivative for expression of FlmD with D55A mutation	This study
pET28a-*flmDD12K*	pET28a derivative for expression of FlmD with D12K mutation	This study
pBT	Bacterial two-hybrid bait plasmid with λ repressor protein (λcI)	Stratagene
pBT-*cheA*	pBT derivative with λcI linked to CheA	[7]
pBT-*flmDD12K*	pBT derivative with λcI linked to FlmDD12K	This study
pBT-*flmDD55A*	pBT derivative with λcI linked to FlmDD55A	This study
pTRG	Bacterial two-hybrid bait plasmid with α-subunit of RNA polymerase (RNAp)	Stratagene
pTRG-*cheW*	pTRG derivative with RNAp linked to CheW	[7]
pTRG-*soxR*	pTRG derivative with RNAp linked to SoxR	This study

**Table 2:** Genes that show significant and >2-fold change at the transcriptional level in *C. testosteroni* strain CNB-1Δ*flmD*.

Gene	Locus_Tag	Annotations	Padj	log2FoldChange
*hmp*	CtCNB1_0015	ferredoxin	2.39 × 10^2^	1.1233
*boxA*	CtCNB1_0065	benzoyl-CoA oxygenase	3.01 × 10^3^	1.5735
*boxB*	CtCNB1_0066	benzoyl-CoA oxygenase	3.81 × 10^13^	1.6828
*boxC*	CtCNB1_0067	benzoyl-CoA-dihydrodiol lyase	3.08 × 10^6^	1.2693
*livK1*	CtCNB1_0096	ABC transporter permease	6.45 × 10^7^	1.249
*bCL*	CtCNB1_0097	4-hydroxybenzoate--CoA ligase	2.09 × 10^6^	1.1543
*tauA*	CtCNB1_0160	ABC transporter permease	1.48 × 10^9^	1.2986
*tauC*	CtCNB1_0161	ABC transporter permease	8.38 × 10^4^	1.1119
*acrA*	CtCNB1_0177	RND family efflux transporter MFP subunit	9.97 × 10^8^	1.0644
*tolC*	CtCNB1_0179	RND transporter	1.74 × 10^6^	1.2098
CtCNB1_0381	CtCNB1_0381	hypothetical protein	4.06 × 10^3^	1.108
*caiD*	CtCNB1_0392	enoyl-CoA hydratase	1.77 × 10^7^	1.0308
*gpmB*	CtCNB1_0417	phosphoglycerate mutase	2.67 × 10^2^	1.0422
*dhlC*	CtCNB1_0516	SSS sodium solute transporter superfamily protein	2.94 × 10^4^	1.1811
*araJ*	CtCNB1_0518	major facilitator transporter	3.17 × 10^4^	1.4008
*ansP*	CtCNB1_0951	proline-specific permease	5.09 × 10^5^	1.6276
*dadA*	CtCNB1_0952	amino acid dehydrogenase	4.35 × 10^4^	1.0916
*livK2*	CtCNB1_1147	twin-arginine translocation pathway signal protein	1.94 × 10^3^	1.2937
*eutG*	CtCNB1_1590	4-hydroxybutyrate dehydrogenase	2.33 × 10^3^	1.1806
CtCNB1_2363	CtCNB1_2363	hypothetical protein	1.15 × 10^2^	1.0003
*soxX*	CtCNB1_2868	SoxX protein	3.36 × 10^2^	1.0829
*soxA*	CtCNB1_2869	SoxA protein	4.58 × 10^3^	1.0581
*soxY*	CtCNB1_2871	twin-arginine translocation pathway signal	2.94 × 10^4^	1.1995
*soxD*	CtCNB1_2872	cytochrome C	2.07 × 10^5^	1.3067
CtCNB1_3408	CtCNB1_3408	hypothetical protein	2.02 × 10^2^	1.1134
*dctP*	CtCNB1_3427	TRAP dicarboxylate transporter, DctP subunit	2.54 × 10^18^	1.1641
*ilvD*	CtCNB1_3428	phosphogluconate dehydratase	3.83 × 10^11^	1.1086
*eda*	CtCNB1_3429	keto-deoxy-phosphogluconate aldolase	2.26 × 10^7^	1.4534
*livK3*	CtCNB1_3479	ABC transporter permease	2.97 × 10^3^	1.097
*sapF*	CtCNB1_3509	ABC-type antimicrobial peptide	1.39 × 10^3^	1.2249
*coxL*	CtCNB1_3514	carbon monoxide dehydrogenase	9.86 × 10^3^	1.1075
*coxS*	CtCNB1_3515	carbon monoxide dehydrogenase	3.64 × 10^2^	1.079
*ilvB*	CtCNB1_4060	thiamine pyrophosphate-requiring enzymes	3.64 × 10^3^	1.0155
*apbA*	CtCNB1_4061	2-dehydropantoate 2-reductase	1.80 × 10^2^	1.4053
*tctC*	CtCNB1_4499	ABC transporter substrate-binding protein	9.44 × 10^12^	1.3628
CtCNB1_4500	CtCNB1_4500	hypothetical protein	1.07 × 10^5^	1.6667
*ubiH*	CtCNB1_4501	FAD-dependent oxidoreductase	5.75 × 10^4^	1.214
*thiJ*	CtCNB1_4569	putative intracellular protease/amidase	2.03 × 10^14^	1.0617
*modA*	CtCNB1_4661	extracellular solute-binding protein	5.80 × 10^5^	1.1208

**Table 3:** Genes that show significant and >2-fold change at the transcriptional level in *C. testosteroni* strain CNB-1/Ov*flmD*.

Gene	Locus_Tag	Annotations	Padj	log2FoldChange
*tolC*	CtCNB1_0179	RND transporter	5.23 × 10^3^	−1.2708
CtCNB1_0378	CtCNB1_0378	hypothetical protein	2.67 × 10^3^	−1.6453
CtCNB1_0381	CtCNB1_0381	hypothetical protein	1.12 × 10^4^	−1.6966
CtCNB1_0383	CtCNB1_0383	hypothetical protein	4.46 × 10^3^	−1.3307
*dhlC*	CtCNB1_0516	SSS sodium solute transporter superfamily	4.24 × 10^2^	−1.4034
*ansP*	CtCNB1_0951	proline-specific permease	1.10 × 10^3^	−1.3189
*cirA*	CtCNB1_1178	TonB-dependent receptor	6.02 × 10^10^	−1.132
*nrdA*	CtCNB1_1179	ribonucleotide reductase	2.33 × 10^2^	−2.1049
*hemS*	CtCNB1_1180	ribonucleotide reductase	3.54 × 10^20^	−1.5981
*chuT*	CtCNB1_1181	ABC transporter substrate-binding protein	2.26 × 10^13^	−1.5584
*fepD*	CtCNB1_1182	hemin transport system permease protein	2.71 × 10^2^	−1.3963
*fepC*	CtCNB1_1183	ABC transporter	4.39 × 10^4^	−1.2717
*fhuE*	CtCNB1_1601	ligand-gated channel protein	1.73 × 10^3^	−1.2529
*cbiK*	CtCNB1_1602	TonB-dependent receptor	2.04 × 10^2^	−1.2527
CtCNB1_1891	CtCNB1_1891	hypothetical protein	1.77 × 10^19^	1.7244
CtCNB1_1892	CtCNB1_1892	hypothetical protein	4.26 × 10^5^	1.2684
CtCNB1_2304	CtCNB1_2304	hypothetical protein	6.55 × 10^3^	1.1858
*oafA*	CtCNB1_3421	acyltransferase	3.30 × 10^4^	−1.3331
*coxG*	CtCNB1_3510	carbon monoxide dehydrogenase subunit G	4.97 × 10^2^	−1.2019
*hppD*	CtCNB1_3836	4-hydroxyphenylpyruvate dioxygenase	3.02 × 10^13^	−1.2022
CtCNB1_3837	CtCNB1_3837	hypothetical protein	6.02 × 10^10^	−1.0953
*lraI*	CtCNB1_3842	ABC-type metal ion transport system	4.71 × 10^2^	−1.462
CtCNB1_3869	CtCNB1_3869	hypothetical protein	6.64 × 10^4^	−1.3152
*fepA*	CtCNB1_4259	TonB-dependent receptor	6.55 × 10^3^	−1.0318
*livK1*	CtCNB1_4328	ABC transporter permease	5.94 × 10^17^	−1.2227
*livK2*	CtCNB1_4497	ABC transporter permease	2.55 × 10^6^	−1.2985

## Data Availability

The RNA-Seq data are submitted to the NCBI BioProject database (Project Accession No: PRJNA393537). All other data generated or analyzed during this study are included in this published article (and its Supplementary Information Files).

## Supplementary Materials

The following supporting information can be downloaded at: https://www.mdpi.com/article/10.3390/microorganisms10020356/s1, Figure S1: The growth of *acrAB-tolC* operon mutant strains of *C. testosteroni*; Figure S2: The multiple sequence alignment of receiver domains; Figure S3. The effect of FlmD variant proteins on the interaction between SoxR and DNA.
