# Supplementary material for: The Response Regulator FlmD Regulates Biofilm Formation in Comamonas testosteroni through the Transcriptional Activator SoxR
Source: Microorganisms. 2022 Feb 4;10(2):356. doi: 10.3390/microorganisms10020356 (PMC8880074; doi:10.3390/microorganisms10020356)
Supplement: Supplementary file 1 [file microorganisms-10-00356-s001.zip › microorganisms-1548085-Supplementary.pdf]

microorganisms

MDPI

Supplementary Matereials

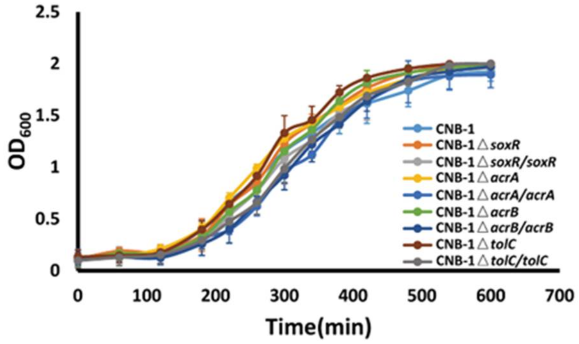

Figure S1. The growth of *acrAB-tolC* operon mutant strains of *C. testosteroni*. The deletion of *acrAB-tolC* operon genes has no effect on growth.

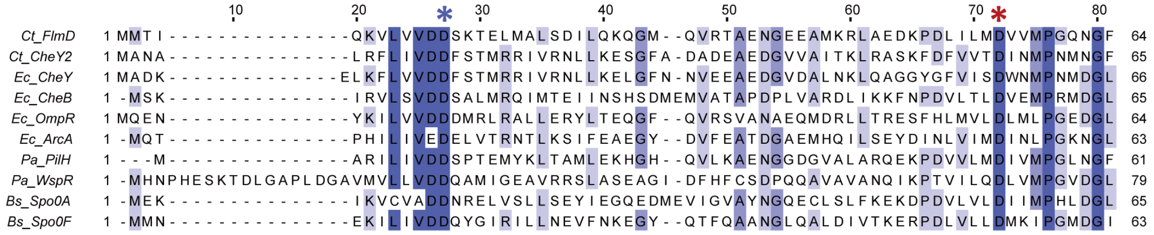

Figure S2. The multiple sequence alignment of receiver domains. Identification of amino acid residues related to response regulator phosphorylation by multiple sequence alignment of receiver domains. Ct: *Comamonas testosteroni*; Ec: *Escherichia coli*; Pa: *Pseudomonas aeruginosa*; and Bs: *Bacillus subtilis*. The red asterisk indicates conserved residues, which is the phosphorylation site of response regulators. The blue asterisk indicates conserved residues, where mutation from an aspartate to a lysine residue is equivalent to a phosphorylated response regulator.

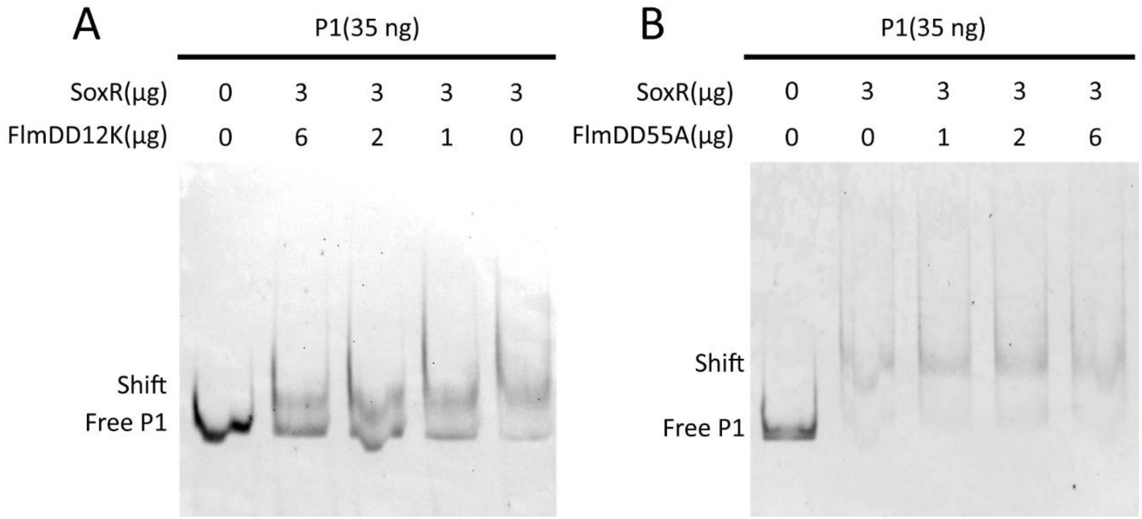

Figure S3. The effect of FlmD variant proteins on the interaction between SoxR and DNA. Changes in the interaction between SoxR and the *acrAB-tolC* operon on the addition of FlmD variants to gel shift assay systems are shown. The effect of FlmD(D12K) is concentration-dependent (A), while FlmD(D55A) has no effect (B).
